# Redox Homeostasis in Cardiovascular Disease: The Role of Mitochondrial Sirtuins

**DOI:** 10.3389/fendo.2022.858330

**Published:** 2022-03-18

**Authors:** Alberto Zullo, Rosa Guida, Rosaria Sciarrillo, Francesco P. Mancini

**Affiliations:** ^1^ Department of Sciences and Technologies, University of Sannio, Benevento, Italy; ^2^ CEINGE Advanced Biotechnologies s.c.a.r.l., Naples, Italy; ^3^ Clinical Scientific Institutes Maugeri IRCCS, Cardiac Rehabilitation Unit of Telese Terme Institute, Telese Terme, Italy

**Keywords:** mitochondria, sirtuins, ROS, energy metabolism, cardiovascular disease

## Abstract

Cardiovascular disease (CVD) is still the leading cause of death worldwide. Despite successful advances in both pharmacological and lifestyle strategies to fight well-established risk factors, the burden of CVD is still increasing. Therefore, it is necessary to further deepen our knowledge of the pathogenesis of the disease for developing novel therapies to limit even more its related morbidity and mortality. Oxidative stress has been identified as a common trait of several manifestations of CVD and could be a promising target for innovative treatments. Mitochondria are a major source of oxidative stress and sirtuins are a family of enzymes that generate different post-translational protein modifications, thus regulating important cellular processes, including cell cycle, autophagy, gene expression, and others. In particular, three sirtuins, SIRT3, SIRT4, and SIRT5 are located within the mitochondrial matrix where they regulate energy production and antioxidant pathways. Therefore, these sirtuins are strongly involved in the balance between oxidant and antioxidant mechanisms. In this review, we summarize the activities of these sirtuins with a special focus on their role in the control of oxidative stress, in relation to energy metabolism, atherosclerosis, and CVD.

## Introduction

Cardiovascular disease (CVD) is still the leading cause of death worldwide, accounting for almost 18 million victims in 2019 (1). The social and economic cost of CVD is constantly rising, also due to the increase of elderly population and the spread of metabolic disorders, known risk factors for CVD. CVD comprises conditions that affect the heart or blood vessels, such as coronary heart disease, stroke, peripheral arterial disease congestive heart failure, heart arrhythmia, congenital heart disease, endocarditis, and aortic disease (1).

The risk factors of CVD are various and include both genetic and non-genetic determinants. Besides the most acknowledged risk factors that affect mainly atherosclerotic CVD (ASCVD), such as hypercholesterolemia, hypertension, smoking, diabetes, physical inactivity, overweight/obesity, family history of CVD, and ethnic background, oxidative stress has been identified as a common denominator of several types of CVD (2). Oxidative stress originates in cells and tissues when reactive oxygen species (ROS) accumulate, due to an imbalance between ROS production and antioxidant defenses. ROS are produced mainly by mitochondria, whose activity is modulated *via* a multitude of molecules, including sirtuins. These evolutionary conserved proteins are at the crossroad of important cellular processes, and favor health and longevity also in humans. Three sirtuins, namely SIRT3, 4, and 5 are localized within mitochondrial matrix where they control energy metabolism and ROS homeostasis.

This review aims at assessing the role of mitochondrial sirtuins in the ROS contribution to the onset and progression of CVD.

## ROS Metabolism

ROS are very unstable molecules and comprise oxygen free radicals (superoxide, hydroxyl radicals, and peroxyl radicals), and non-radicals (hydrogen peroxide, and hypochlorous acid) (3). Because of their instability, ROS are highly reactive and, therefore, can react with and damage proteins, lipids, and DNA (4).

**Figure 1:**
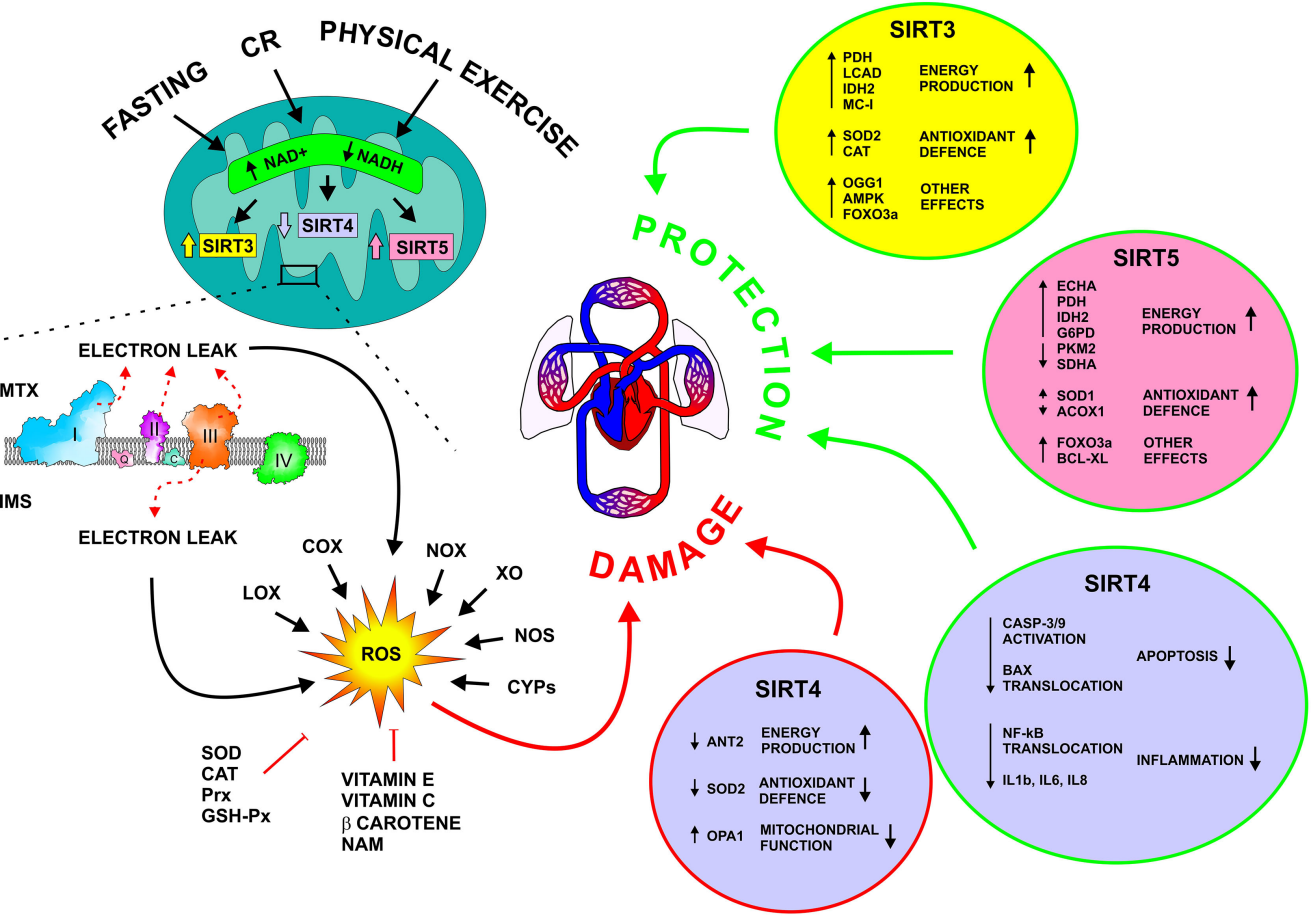
Molecular mechanisms underlying ROS production and the activity of mitochondrial sirtuins on CVD. SIRT3, 4, and 5 control cellular metabolism, and mitochondrial and ROS homeostasis. Dietary interventions, in the forms of fasting and calorie restriction, as well as physical exercise, stimulate SIRT3 and 5 and repress SIRT4. SIRT3 and 5 activate energy production and antioxidant pathways, resulting in the protection of the cardiovascular system, particularly during metabolic and oxidative stress. Differently, SIRT4 seems to have both a detrimental and a beneficial effect on the cardiovascular system. ACOX1, Acyl-CoA Oxidase 1; AMPK, AMP-Activated Protein Kinase; ANT2, Adenine Nucleotide Translocator 2; Bcl-XL, B-cell lymphoma extra-large; CASP-3/9; Caspase 3 and 9; CR, Calorie Restriction; COX, Cytochrome C Oxidase; CYPs, Cytochrome P450; ECHA, Enoyl-Coenzyme A Hydratase; FOXO2A, Forkhead Box O3A; G6PD, Glucose-6-Phosphate Dehydrogenase; GSH-Px, Glutathione peroxidase; IDH2, Isocitrate Dehydrogenase 2; IL1b, Interleukin 1 beta; IL6, Interleukin 6; IL8, Interleukin 8; IMS, intermembrane space; LCAD, Long-Chain Acyl-CoA Dehydrogenase; LOX, Lipoxygenase; MC-I, Mitochondrial Complex I; mTOR, Mammalian Target Of Rapamycin; MTX, Mitochondrial Matrix; NAM, Nicotinamide; NF-kB, Nuclear factor-kappa b; NOS, Nitric Oxide Synthases; NOX, NADPH Oxidase; OGG1, 8-oxoguanine-DNA glycosylase; OPA1, Optic Atrophy 1; Prx, Peroxiredoxin; PDH, Pyruvate Dehydrogenase; PKM2, Pyruvate Kinase M1/2; ROS, Reactive oxygen species; SDHA, Succinate Dehydrogenase Complex, subunit A; SOD, Superoxide Dismutase; XO, Xanthine oxidase.

ROS are produced mainly by mitochondria along the respiratory chain, but other organelles, such as the peroxisome and endoplasmic reticulum may contribute to ROS formation. Several intra [like α-glycerophosphate-, α-ketoglutarate-, pyruvate dehydrogenases, cytochrome P450 enzymes (CYPs), nitric oxide synthases (NOS)] and extra-mitochondrial enzymes, primarily NADPH oxidases (NOX), but also xanthine oxidase (XO), NOS, cyclooxygenases (COX), CYPs, and lipoxygenases (LOX), can generate ROS (**Figure 1**) (5, 6) Besides endogenous processes, ROS levels can be increased also by exogenous sources including pollutants, radiations, cigarette smoking, xenobiotics, certain foods and drugs (7). To counteract the production of ROS, numerous endogenous and exogenous antioxidant factors are present within the cells. Among endogenous antioxidants there are enzymes, like superoxide dismutase (SOD), catalase (CAT), peroxiredoxin (Prx), and glutathione peroxidase (GSH-Px), and compounds, particularly reduced glutathione (GSH). Exogenous antioxidants comprise mainly vitamins and pro-vitamins, namely vitamin E, β-carotene, ascorbate, and nicotinamide (NAM) (**Figure 1**) (8).

Although excessive ROS accumulation is associated with many diseases, including CVD, controlled and limited amounts of ROS are important mediators involved in different cellular processes, such as metabolism, immunity, growth, and differentiation (4, 9). Therefore, it is essential for the cell to strictly regulate ROS levels by balancing ROS production and scavenging.

ROS production is strictly linked to energy metabolism. Indeed, increased energy demand needs enhanced ATP synthesis, which, in turn, requires increased activity of the mitochondrial respiratory chain, thus causing augmented electron leak and consequent production of ROS (**Figure 1**) (4).

Mitochondria produce ATP through the oxidation of different substrates, such as amino acids, lipids, and carbohydrate derivatives, the transfer of electrons through the respiratory chain, also called electron transport chain (ETC), the generation of the proton gradient in the intermembrane space, and the activity of the ATP synthase.

Although ATP generation within mitochondria is very efficient, electrons leak from the ETC and give rise to about 90% of the cellular ROS.

## ROS And Atherosclerosis

Excessive ROS generation drives mitochondrial damage, which, in endothelial, and cardiac muscle cells, leads to cellular dysfunction, thus favoring the development of ASCVD as well as other heart diseases like hypertrophic and dilated cardiomyopathy, heart failure, and impaired cardiac activity due to ischemia/reperfusion (I/R) injury (10, 11).

In the last hundred years, it became clear that CVD, and particularly ASCVD, is mainly a metabolic disease (12). Indeed, abnormalities of lipid, glucose, salt, energy, and ROS metabolism are major drivers of atherosclerosis, the culprit pathogenic process underlying the vast majority of CVD. All these alterations promote the formation of the atherosclerotic plaque within the arterial wall, which progressively obstruct the vessel lumen with consequent ischemia of the downstream tissues. When oxygen supply drops below a threshold value, ischemic tissues become necrotic tissues thus causing severe conditions such as myocardial infarction or stroke. These often-fatal events generally take place when a thrombus originates on top of the atherosclerotic plaque in the advanced stages of the disease and causes complete or almost complete obstruction of the vessel lumen (13). Interestingly, the clustering of metabolic disturbances as the primary inducer of CVD received universal recognition at the end of the last century, when the term “metabolic syndrome” was coined to identify the grouping of metabolic abnormalities, including insulin resistance, obesity, dyslipidemia, and hypertension, with associated increased risk of CVD (12).

Therefore, a good metabolic control is a strong protection for both vascular and cardiac health.

## Mitochondrial Sirtuins

The survival of the living organisms relies on the ability to adapt to environmental changes which require constant metabolic rewiring. Nutrient availability is a major variable among environmental conditions. Organisms have developed complex mechanisms for adapting to changes in energy demand, energy supply, and nutrient availability (14). Mitochondria are the core of this bioenergetic adaptation, and several players have been identified that control these homeostatic mechanisms. Among these, sirtuins are a family of enzymes, that orchestrate cellular metabolism and response to changes in nutrient availability. Sirtuins comprise evolutionarily conserved proteins with different nicotinamide dinucleotide (NAD)^+^-dependent enzymatic activities such as deacetylase, desuccinylase, demaloynylase, deglutarylase, long-chain deacylase, lipoamidase, and ADP-ribosyltransferase. In particular, deacetylation is common to all the isoforms, although with a variable degree (15).

In mammalian cells, seven types of sirtuins (SIRT1-7) regulating essential processes, like lifespan prolongation, cell cycle, autophagy, gene expression, DNA repair, metabolism, and stress resistance, have been identified. Sirtuins have different cellular locations, and some of them are present in more than one cellular compartment (16, 17). In particular, three sirtuins, SIRT3, SIRT4, and SIRT5 reside inside the mitochondrial matrix where they regulate proteins involved in metabolic reactions, energy production, antioxidant pathways, apoptosis, and autophagy, thus promoting mitochondrial homeostasis (18, 19). These targets, including SOD2, FOXO, p53, AMP-activated protein kinase (AMPK), and mammalian target of rapamycin (mTOR) are post-translationally modified by sirtuins, and allow a rapid homeostatic metabolic response to changes in energy expenditure/supply ratio, which occurs in several circumstances, such as calorie restriction (CR), fasting, physical exercise, and metabolic stress (**Figure 1**) (18, 20, 21).

CR is a dietary regimen consisting of a 30% - 50% reduction of the regular calorie intake, without malnutrition (22). Fasting diet is an eating pattern in which the food intake is strictly time-limited (23). Nutritional interventions, in the form of fasting diets and CR, have been shown to improve human health (23).

The expression and activity of mitochondrial sirtuins are strictly related to the metabolic state of the cell and allow metabolic adaptation, which consists of rebalancing the equilibrium between production and consumption of ATP during negative energy balance (22). Metabolic adaptation enhances metabolic efficiency and lowers energy expenditure (24–27).

Reduced nutrient availability enhances SIRT3 and SIRT5, dampens SIRT4 activity, and correspondingly nutrient excess leads to a reduction of SIRT3 and SIRT5, and an increase of SIRT4 activity (18).

Under fasting conditions, mitochondrial sirtuins can activate cellular defense mechanisms and compensate for the lack of energy sources; nutrient excess, as well as metabolic diseases, can dysregulate sirtuins activity and trigger a vicious cycle leading to cell death and organ dysfunction (18, 28).

NAD^+^ is a central coenzyme in many metabolic pathways, particularly glycolysis, fatty acid oxidation (FAO), and oxidative phosphorylation (OXPHOS), by shuttling electrons for ATP production. Its reduced form, NADH, is the first electron donor of the respiratory chain. NAD^+^ can be synthesized from tryptophan, nicotinic acid, NAM, and NAM riboside (29). Moreover, the intracellular concentration of this cofactor rises at decreasing ratios between energy supply and energy expenditure, i.e. during physical exercise, fasting, and CR (30).

Interestingly, NAD^+^ also modulates the activity of several enzymes within the cells, including sirtuins, which are stimulated by increased cellular levels of this coenzyme (31). This makes sirtuins cellular energy-sensing proteins.

Given their function, sirtuins are ubiquitously expressed in human tissues. It is not surprising that impairment of sirtuin function has been associated with diseases and aging. Moreover, an adequate activity of these molecules helps organisms to maintain a healthy condition and increases their lifespan (32, 33). Aging, as well as obesity and lack of regular physical exercise, are associated with decreased cellular NAD^+^ concentrations and sirtuin activity (34).

Because of the central role of sirtuins in regulating metabolism and promoting health and longevity in humans, this protein family is attracting a lot of scientific interest. The main activities of mitochondrial sirtuins relating to metabolic regulation, redox balance, and CVD are reported in the **Table 1**.

**Table 1:** Main activities of mitochondrial sirtuins relating to metabolic regulation, redox balance and CVD.

	Expression	Metabolic regulation	Redox balance	CV effects
SIRT3	**↑** by reduced nutrient availability	**↑** OXPHOS, TCA, FAO, glucose utilization	**↓** ROS **↑** SOD2, CAT	Protects from cardiac hypertrophy, pulmonary hypertension, I/R injury, hypertensive/obesity-dependent cardiac remodeling, diabetic cardiomyopathy, T2D, aortic dissection, foam cell formation, ROS-induced EPC damage
SIRT4	**↓** by reduced nutrient availability	**↑** mitochondrial fusion **↓** mitophagy, insulin secretion, and FAO	**↑** ROS **↓** SOD2	Induction of ANG-2 dependent heart failure, fibrosis, and remodeling. Protection from hypoxic insult.
SIRT5	**↑** by reduced nutrient availability	**↑** OXPHOS, TCA, FAO, glucose utilization, ketone body formation	**↓** ROS **↑** GSH, SOD1 **↓** ACOX1	Protects from I/R injury, oxidative stress-induced cardiomyocyte apoptosis, aging-associated cardiac dysfunction and hypertrophy

ACOX1, acyl-CoA oxidase 1; ANG-2, Angiopoietin 2; CAT, Catalase; EPC, Endothelial Progenitor Cell; FAO, Fatty Acid Oxidation; GSH, Glutathione; I/R, Ischemia/Reperfusion; OXPHOS, Oxidative Phosphorylation; ROS, Reactive Oxygen Species, SOD, Superoxide dismutase; TCA, Tricarboxylic Acid Cycle; T2D, Type2 Diabetes.

## SIRT3, ROS, and CVD

SIRT3 regulates major mitochondrial functions such as OXPHOS, tricarboxylic acid (TCA) cycle, and FAO by deacetylating and activating pyruvate dehydrogenase (PDH), long-chain acyl-CoA dehydrogenase (LCAD), and isocitrate dehydrogenase 2 (IDH2). The latter is deacetylated at the K413 position. Moreover, this sirtuin hinders the production of ROS by activating ROS detoxifying enzymes, thus protecting mitochondria and the whole cell from the detrimental effects of oxidative stress. At a molecular level, SIRT3 directly activates SOD2 by deacetylating specific lysine residues (K53, K89, K68, and K122) (10, 27). Additionally, SIRT3, by targeting the transcription factor forkhead box O3a (FOXO3a), induces the transcription of SOD2, and catalase (CAT) (10).

Moreover, SIRT3-activated IDH2 drives the mitochondrial IDH2-NADPH-GSH antioxidant system (11). Further protection from ROS-induced damages is provided by the contribution of SIRT3 to DNA repair by deacetylating 8-oxoguanine-DNA glycosylase 1 (OGG1) (27). Finally, SIRT3 regulates also proton flux management, which is also closely linked to ROS generation (35).

On this basis, it is no surprise that lack of SIRT3 activity results in cellular dysfunction and is associated with the development of several diseases, among which metabolic disorders, aging, and CVD (10, 18, 27). Cardiomyocytes are particularly sensitive to the absence or reduction of SIRT3 activity because they have plenty of mitochondria due to their requirement of large amounts of ATP to support their continuous contractile activity (11). Reduction of SIRT3 activity in cardiomyocytes leads to increased hyperacetylation and inhibition of mitochondrial enzymes involved in bioenergetic pathways and to increased ROS generation, with harmful consequences for cardiac function (11). SIRT3 can preserve cardiac function through the activation of the AMPK/mTOR, FOXO3a, Pink1/Parkin pathways, and SOD2 (11).

The role of SIRT3 in the context of CVD has been widely studied employing SIRT3-Knock Out (KO) animal models. In SIRT3-KO mice, the mitochondrial oxidative capacity is altered and the H_2_O_2_ production is increased (36). Mice lacking SIRT3 developed cardiac hypertrophy and pulmonary hypertension due to the opening of the permeability transition pore and the increased activity of the transcription factors HIF1a, STAT3, and NFATc2, respectively (10, 37).

Studies *in vitro* and in aged and SIRT3-KO mice demonstrated that reduced SIRT3 activity in mitochondria decreases the cellular recovery from I/R insult. In particular, the authors showed that the activity of two SIRT3 targets, i.e. mitochondrial complex I and SOD2, were inhibited (38). Accordingly, some reports indicate that, after I/R, the increase of ROS production in mitochondria from SIRT3-deficient cardiomyocytes is associated with a reduction in SOD activity, but not with oxidative damage to mitochondrial proteins and DNA (39).

The protective role of SIRT3 in hypertensive cardiac remodeling has been linked to the activity of this sirtuin on mitochondria and angiogenesis in angiotensin 2- (ANG2) treated mice. SIRT3 expression in endothelial cells is also fundamental for limiting the negative effects of cardiac remodeling (40). SIRT3 protects the cardiovascular system also by regulating glucose metabolism, optimizing the exploitation of the different sources of energy, and reducing ROS production at the same time. Studies on mouse cardiomyocytes showed that the harmful effects of high-glucose treatment were exacerbated by SIRT3 deficiency. In particular, the mitochondrial membrane potential collapsed, and the level of ROS increased, thus leading to cell death (41). Moreover, H9C2 cardiomyoblasts exposed to high glucose activated apoptosis, and diabetic mice developed microvascular rarefaction, cardiac fibrosis, hypertrophy, and dysfunction. Interestingly, SIRT3 overexpression both *in vitro* and *in vivo* could rescue the molecular, structural and functional cardiac impairment following the hyperglycemic conditions (28). Furthermore, it has been recently suggested that lack of SIRT3 activity may affect glucose homeostasis and favor the onset of insulin resistance and type 2 diabetes in humans (27).

Also in endothelial cells, SIRT3 is indispensable for glycolytic metabolism. Indeed, the lack of SIRT3 expression led to reduction of glycolysis and increase in mitochondrial respiration with a consequential increase in ROS production (42).

The connection between SIRT3, ROS, and CVD is further highlighted in the presence of altered lipid metabolism, as it occurs when obesity develops. It is well known that obesity is associated with several cardiovascular complications, including cardiac remodeling and dysfunction. The development of inflammatory and fibrotic processes drives pathological remodeling of tissues, leading to organ dysfunction (43).

In obesity, NACHT, LRR, and PYD Containing Protein 3 (NLRP3) inflammasome is responsible for the induction of inflammatory processes in endothelial cells (44). Based on recent studies, NLRP3 is repressed by SOD2. Inactivation of the SIRT3-SOD2 axis within mitochondria has been demonstrated to induce vascular inflammation through the NLRP3 inflammasome, as reported in *in vitro* studies on human umbilical vein endothelial cells (HUVECs). In addition, stimulation of SIRT3, for example by prolonged fasting, suppressed NLRP3 inflammasome *via* SOD2 activation (45).

Hearts of mice under a high-fat diet (HFD) exhibited reduced expression of SIRT3, increased ROS, activation of the inflammatory and fibrotic processes, and cardiac dysfunction. Moreover, the lack of SIRT3 exacerbated HFD-dependent cardiac remodeling by affecting ROS, nuclear factor-kappa b (Nf-κB), monocyte chemoattractant protein-1 (MCP-1), and hypoxia-inducible factor (HIF) signaling pathways (46, 47).

Besides endothelial cells and cardiomyocytes, recent experimental evidence demonstrated that also vascular smooth muscle cells (VSMCs) benefit from SIRT3 expression. Indeed, in SIRT3-KO mice, the risk for thoracic aortic dissection is increased, due to the lack of SIRT3-dependent antioxidative, antiapoptotic, and anti-inflammatory activity on VSMCs (48).

In VSMCs, the Toll-Like Receptor 4 (TLR4)-dependent downregulation of SIRT1 and SIRT3 mediated the formation of foam cells induced by oxidized low density lipoproteins (oxLDL). The levels of ROS and foam cell-like markers were inversely correlated with the expression of the sirtuins (49).

Also, CR relies on the activity of SIRT3 for boosting antioxidative reactions (10).

As far as autophagy, SIRT3 has a controversial role. Indeed, SIRT3 can both enhance and attenuate autophagy. Given the role of autophagy in ischemic injury, it has been speculated that SIRT3 modulation could be a therapeutic strategy to reduce the damages of myocardial ischemia (50).

Impaired mitochondrial activity affects also aging (11). Moreover, SIRT3 deficiency contributes to myocardial I/R injury in aged mice. During aging, I/R stress induces excessive ROS production, which, in turn, damages mitochondria and impairs cardiac cell viability (11).

In the context of regenerative medicine, it has been demonstrated that SIRT3 is critical for the use of cellular therapy in myocardial infarction (51). In particular, in bone marrow-derived endothelial progenitor cells, the lack of SIRT3 enhances ROS formation and reduces their angiogenic potential (51).

## SIRT4, ROS, and CVD

SIRT4 is mainly active in the mitochondrial matrix where it functions as a protein lipoamidase, ADP-ribosyl transferase, and deacetylase. SIRT4 has tumor-suppressing activity by interfering with cyclin/cyclin-dependent kinase (CDK) mechanisms, similarly to CDK inhibitors (52). In addition, SIRT4 has other important biological roles, such as controlling metabolism, energy production, and antioxidant activity (53, 54). This sirtuin is expressed in several different tissues, among which heart and endothelial cells (27, 55, 56).

The expression of SIRT4 in mammalian cells is associated with ATP production. The molecular mechanism relies on the deacetylation of mitochondrial adenine nucleotide translocase 2 (ANT2), which allows ADP and ATP to move in or out of the mitochondria, respectively. In the context of the cardiovascular system, SIRT4 has a controversial role, i.e. it can be either protective or harmful, depending on the context. This sirtuin induces heart failure, fibrosis, and hypotrophy in ANG2-treated mice. In SIRT4-transfected HEK293 cells and fibroblasts, SIRT4 promotes the fusion of mitochondria, hinders mitophagy *via* interaction with optic atrophy 1 (OPA1) protein, and enhances the accumulation of ROS by interacting with SIRT3, thus preventing its activation of SOD2 (**Figure 1**) (27).

In SIRT4-KO mice, SIRT4 upregulation worsens the ANG2-dependent increase in ROS and pathological heart remodeling (57).

Interestingly, SIRT4 is protective in hypoxic and some atherogenic and inflammatory conditions. Indeed, hypoxia led to a reduction in SIRT4 expression both in H9C2 cardiomyoblasts and in HUVEC (56, 58). This downregulation has been implicated in apoptotic cell death, mitochondrial uncoupling, and endothelial dysfunction (56, 58). At the molecular level, SIRT4 inhibited the activation of caspase 3 and 9, and decreasd Bax translocation to mitochondria (58).

Similarly, oxLDL treatment of HUVEC reduced SIRT4 expression and induced apoptotic cell death (55, 59). As for inflammation, lipopolysaccharide and oxLDL treatments of HUVEC, inhibited SIRT4, increased NF-κB translocation, and enhanced the production of the pro-inflammatory cytokines IL-1β, IL-6 and IL-8 (55).

Consistently, the overexpression of SIRT4 reduced the deleterious effect of the hypoxic and atherogenic/inflammatory treatments (55, 58, 59).

## SIRT5, ROS, and CVD

SIRT5 is a mitochondrial sirtuin with deacetylase (weak), desuccinylase, demalonylase, and deglutarylase activity on mitochondrial proteins (27, 53).

Many effects of SIRT5 are similar to those of SIRT3. Indeed, SIRT5 regulates protein of the glycolysis, TCA cycle, FAO, ETC, ketone body formation, ROS generation, and detoxification.

In particular, it targets succinate dehydrogenase complex, subunit A (SDHA), PDH, IDH2, and glucose-6-phosphate dehydrogenase (G6PD), whose activity can influence the intracellular ROS levels. SIRT5 removes succinyl and glutaryl groups from IDH2, and G6PD, thus increasing NADPH production. NADPH reduces cellular ROS, *via* the reduction of oxidized glutathione (GSSG) into glutathione (GSH). Consistently, SIRT5-KO mice have increased ROS levels (60).

Besides its mitochondrial location, SIRT5 has been detected also in the cytosol and in peroxysomes, where it desuccinylates and activates SOD1 and inhibits pyruvate kinase M1/2 (PKM2), and peroxisomal acyl-CoA oxidase 1 (ACOX1), thus attenuating the oxidative stress (**Figure 1**) (27).

Similar to SIRT3, SIRT5 protects cells from stressors by activating homeostatic mechanisms (61).

With regard to CVD, it is notable that SIRT5 is upregulated in cardiac cells also under normal physiological conditions, as compared to cells from other tissues (62, 63). Moreover, SIRT5 is upregulated in rat cardiomyocytes during hypoxia (64).

Several pieces of evidence indicated that SIRT5, through the activation of FOXO3a, upregulates the antioxidant genes, and promotes enhanced resistance to oxidative stress, thus preserving cardiomyocyte viability (61).

Lack of SIRT5 in cardiac cells results in reduced desuccinylation of mitochondrial proteins involved in OXPHOS, FAO, ketogenesis, amino acid catabolism, and the TCA cycle (65).

In mouse myocardium, SIRT5 desuccinylates and activates enoyl-coenzyme A hydratase (ECHA), an important enzyme of the FAO. Therefore, SIRT5-KO mice showed altered fatty acid metabolism and reduced ATP production during energy-demanding conditions such as fasting and exercise (66).

Also, SIRT5 plays a pivotal role in protecting the heart from the deleterious effects of I/R injury through the desuccinylation and inhibition of SDH (65).

In the context of cardiac hypertrophy, SIRT5-KO mice undergoing traverse aortic constriction (TAC) showed a more severe cardiac dysfunction and increased mortality, compared to WT mice (67).

Cardiomyocyte protection by SIRT5 has been documented also *in vitro*. SIRT5 prevents oxidative stress-induced apoptotic cell death by increasing the expression of B-cell lymphoma extra-large (Bcl-XL) (68).

As for the role of SIRT5 in aging, it has been reported that SIRT5 deficiency is associated with cardiac dysfunction and hypertrophy in aged mice (66).

Data collected so far demonstrate that the desuccinylating activity of SIRT5 is critical for the regulation of cardiac cell metabolism under metabolic stress conditions (61).

## Discussion

CVD still represents a heavy social and economic burden worldwide, notwithstanding the significant improvements in preventing and treating this condition by using powerful drugs to fight the traditional risk factors. Therefore, a deeper knowledge of the molecular mechanisms of CVD is still needed to reduce its mortality and morbidity. Metabolic disorders and oxidative stress are important determinants for the onset and progression of CVD.

Recently, sirtuins have been recognized to promote health and increase lifespan in several eukaryotes, including humans. Their dysregulation has been associated with CVD and other diseases. In particular, mitochondrial SIRT3, 4, and 5 may influence cardiovascular health by controlling cellular metabolism and ROS homeostasis. SIRT3 and 5 stimulate energy production and antioxidant pathways, resulting in the protection of the cardiovascular system, particularly during metabolic and oxidative stress. Conversely, data on SIRT4 showed contrasting results. On the one hand, SIRT4 seems to have a detrimental effect on the cardiovascular system, mainly *via* increased ROS production; on the other hand, this sirtuin protects cardiac cells from ischemic, and some atherogenic and inflammatory insults.

Furthermore, SIRT4 has a pivotal role in regulating metabolism. This double face of SIRT4 is an intriguing apparent evolutionary paradox and may reflect the complexity of the metabolic control that must integrate varying nutritional statuses and different organ functions.

In the complex path from sirtuins to cardiovascular homeostatic maintenance, ROS and mitochondrial metabolism represent two cardinal hubs. Dietary interventions, in the forms of fasting and CR, have been demonstrated to modulate sirtuins and promote cardiovascular health. Given the limited feasibility of these strategies, it would be highly desirable to translate into clinical practice the large body of evidence about natural or synthetic activators of mitochondrial sirtuins to fight CVD (69). Indeed, several modulators have already been patented and some clinical trials are already completed or still underway (70–72).

In conclusion, this review presents evidence that mitochondrial sirtuins are at the crossroads of several ROS-mediated etiopathogenetic pathways in CVD and support the hypothesis that targeted modulation of mitochondrial sirtuins by activators/inhibitors might offer, in the future, a possible novel nutritional/pharmacological strategy to treat CVD.

## Author Contributions

AZ and FM conceived the idea. AZ and RG wrote the manuscript. FM and RS revised the manuscript. All authors approved the final version of the manuscript.

## Funding

The production of this manuscript has been financially supported by the Department of Sciences and Technologies, University of Sannio, Benevento, Italy.

## Conflict of Interest

The authors declare that the research was conducted in the absence of any commercial or financial relationships that could be construed as a potential conflict of interest.

The reviewer GR declared a shared affiliation with the author AZ to the handling editor at the time of review.

## Publisher’s Note

All claims expressed in this article are solely those of the authors and do not necessarily represent those of their affiliated organizations, or those of the publisher, the editors and the reviewers. Any product that may be evaluated in this article, or claim that may be made by its manufacturer, is not guaranteed or endorsed by the publisher.
